# Predicting VNN resistance in European sea bass using machine learning on high dimensional low sample size data

**DOI:** 10.3389/fbinf.2026.1718386

**Published:** 2026-05-20

**Authors:** Giovanni Faldani, Enrico Rossignolo, Eleonora Signor, Alessio Longo, Sara Faggion, Luca Bargelloni, Matteo Comin, Cinzia Pizzi

**Affiliations:** 1 Department of Information Engineering, University of Padova, Padova, Italy; 2 Department of Comparative Biomedicine and Food Science, University of Padova, Padova, Italy

**Keywords:** high-dimensional, low sample size, machine learning classification, phenotype prediction, seabass, SNP data

## Abstract

Aquaculture is a rapidly growing sector in the global food production chain as a recognized fundamental source of high-quality proteins. One of the crucial tasks in aquaculture is phenotype prediction. While machine learning research has mainly focused on classification tasks on Big Data, in many bioinformatics applications, including aquaculture, the real challenge behind prediction problems is dealing with small sample and high-dimensional data. In such contexts, it is in fact common that the number of genetic features (such as SNPs) far exceeds the sample size. As a test case, this study focuses on the prediction of resistance to Viral Nervous Necrosis(VNN) from a population of European sea bass. We explore a range of machine learning techniques, from established methods such as Support Vector Machines and Gradient Boosting, to increasingly popular Deep Learning Approaches, also including a variant of image-based classification based on Chaos Game Representation. Besides standard training-test partitioning, we also considered a more challenging partition of the dataset that maximize the genomic distance among training and testing set to better reflect the kind of generalization problem encountered in breeding practice due to data scarcity typical of non-model species. Although all the animals belong to the same population, this approach offered the most appropriate way to ensure the procedure was sufficiently challenging given the available data. We assessed the performance of learning approaches in different scenarios, reducing the data dimensionality by selecting SNPs on the basis of functional information. Our experiments confirmed the difficult nature of this association task. However, each tested tool showed promising results in at least one scenario. While predicting disease susceptibility remains a challenging task for breeding programs, within the boundaries of the tested scenarios, our results show that machine learning approaches, combined with a controlled amount of additional functional information, can help mitigate the issues arising from high dimensional, low sample size datasets typical in the study of non-model species.

## 1 Introduction

The study of genotype-phenotype relationship witnesses a growing interest from the research community, aiming at the identification of disease-connected genetic variants. Since most Single Nucleotide Polymorphisms (SNPs), used as markers for specific genomics regions, exert minimal biological effects, the task of identifying those that are actually disease-connected is considered as a challenging task.

Genome-wide association studies (GWAS) play a fundamental role in unraveling the impact of SNPs, improving our understanding of their association to diseases, shedding light on the underlying mechanisms of observed phenotypes (Uffelmann et al., 2021).

Through GWAS, SNPs can be identified as candidate biomarkers, potentially indicating susceptibility to complex diseases. Despite the success of GWAS in pinpointing disease-related SNPs, unique challenges arise, particularly in the context of big genomic data where high-dimensional datasets often feature far more genetic variables than samples (Uppu et al., 2018). A tightly related problem is the phenotype prediction of a disease from this high-dimensional, low-population SNP data.

In fact, in this context, it is common to deal with datasets that are characterized by a number of SNPs that is orders of magnitude bigger than the number of samples (e.g., $10^{6}$ vs. $10^{3}$ ). This unbalance is responsible for the challenges faced in phenotype prediction, as it makes difficult to navigate the high-dimensional feature space in search of biologically relevant SNPs with such a small number of examples.

Aquaculture is a typical context in which the data characterized by high-dimensional feature space and a relative number of individuals that can be analyzed.

Within the Mediterranean aquaculture, the European sea bass is a highly valued species, carrying substantial economic and cultural importance (Vandeputte et al., 2019). In the past 2 decades, global aquaculture production of European sea bass has seen a significant growth, rising from 7,694 tons in 2000 to 299,810 tons in 2021. However, the industry faces increasing challenges from infectious diseases, which threaten both the sustainability of sea bass farming and the health of cultured populations. Viral Nervous Necrosis is a major viral disease impacting global aquaculture, affecting numerous farmed and ecologically vital species. It is the primary viral infectious disease in European sea bass, responsible for 15% of all on-farm disease-related mortality (Muniesa et al., 2020). VNN resistance in European sea bass is characterized by significant additive genetic variation and recently one genomic region has been detected as significantly associated with this trait (Mukiibi et al., 2025), yet the specific causal gene(s) and mutation(s) underlying this resistance remain unknown.

Machine learning provides a versatile and extensive set of techniques suited to tackle the challenges of these high-dimensional low-population SNP datasets. In our study^1^, we selected XGBoost (Chen and Guestrin, 2016), and COMBI (both SVM and Deep Learning versions) (Mieth et al., 2016; 2021) to cover both “classic” machine learning and “deep” learning approaches. Moreover, we designed an *ad hoc* Chaos Game Representation (CGR) adaptation (Jeffrey, 1990) to mapssequences of SNPs into images which are then classified using a Convolutional Neural Network (CNN). Our experiments cover the analysis of very high dimensional datasets on different tissues, as well as the analysis of reduced SNP datasets that consider SNPs in active regions only. For this specific analysis we also selected random sub-samples of SNPs of the same size, as a control test on the efficacy of the selection of SNPs in active regions. We conducted both a traditional analysis with a 80%−20% training/test partitioning of the datasets and a more challenging analysis in which the individuals were partitioned in two clusters based on their genomic relatedness.

In this work, we specifically focus on the challenge of phenotype prediction from SNP data in high-dimensional low sample size settings, using aquaculture genomic data as a case study. Our goal is threefold: (i) to systematically compare classical and deep learning approaches for SNP-based classification, (ii) to investigate the impact of biologically informed SNP selection based on functional genomic annotations, and (iii) to evaluate the effectiveness of alternative data representations, in particular Chaos Game Representation (CGR), in capturing spatial genomic patterns. Additionally, we assess model performance under both standard random splits and more realistic scenarios involving genomically distant populations, providing insights into model generalization in practical breeding contexts.

The paper is organized as follows. In Section 2 we will describe in detail the approaches we used for our analysis and how they were adapted, when needed, for the analysis of SNPs datasets. In Section 3 we will describe the experimental setup and discuss the results of the several classification tests we performed on all the datasets. Finally, we will drive the conclusion of our analysis in Section 4.

## 2 Materials and methods

A SNP (Single Nucleotide Polymorphism) is a variation in the genome of a single nucleotide with respect to the reference genome. SNPs are located within chromosomes and the frequency of variation depends on the frequency of alleles that make up a gene. SNPs act as biological markers and can identify genes associated with a disease. In this paper, we assess different machine learning approaches to predict VNN resistance of about a thousand individuals through the analysis of several SNPs datasets.

To address the phenotype prediction task under a high-dimensional low sample size setting, we employed four complementary machine learning approaches, chosen to cover both classical and deep learning paradigms. First, we used XGBoost, a gradient boosting framework based on decision trees, which iteratively combines weak learners to improve predictive performance. Its built-in regularization and ability to handle sparse, high-dimensional data make it particularly suitable for genomic applications. Second, we adopted the COMBI framework, based on Support Vector Machines (SVM), which integrates statistical feature selection with linear classification. This approach is well-suited for SNP data, as it enables the identification of relevant genetic variants while maintaining model interpretability. Third, we considered DeepCOMBI, an extension of COMBI that replaces the linear classifier with a multilayer perceptron (MLP), allowing the model to capture non-linear relationships among SNPs. This is particularly relevant in genomic contexts where complex interactions between variants may influence the phenotype. Finally, we introduced a novel approach based on Chaos Game Representation (CGR), in which SNP sequences are transformed into image-like representations that preserve their spatial organization along the genome. These representations are then processed using a deep convolutional neural network (CNN), enabling the model to learn higher-order patterns and spatial dependencies that are not captured by traditional feature-based methods.

These four approaches will be presented in the next sections.

### 2.1 XGBoost

XGBoost (eXtreme Gradient Boosting) is a widely used and powerful machine learning algorithm based on the gradient boosting framework (Chen and Guestrin, 2016). It has gained significant popularity due to its efficiency, scalability and effectiveness in a wide variety of data science applications, including bioinformatics and genomics.

In bioinformatics, XGBoost has been applied in tasks such as predicting gene expression values (Li et al., 2019), identifying diseases from biomarkers (Sharma and Verbeke, 2020), and classifying complex biological data, such as those coming from SNP data (Medvedev et al., 2022; Lim et al., 2023). One of the major advantages of XGBoost is its ability to handle sparse data efficiently, which is especially useful when dealing with medical and biological datasets that require data collection, where missing values are common.

**FIGURE 1:**
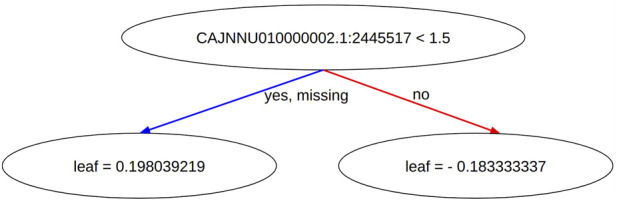
A decision tree is part of the gradient boosting model trained by XGBoost. The figure shows a decision tree where a SNP is used as a decision node. At this node, the value of the SNP determines which branch of the tree to follow. The tree leads to two leaf nodes, each containing a specific value that will be added to the model’s output depending on the SNP’s value.

The model trained with XGBoost can be easily and effectively explained. Gradient boosting is based on decision trees and decision trees themselves are effortlessly interpretable compared to more complex models like neural networks. Each decision tree represents a series of decisions (or splits) based on feature values, and these decisions can be visually examined to understand how the model arrives at its predictions. Operationally this means that a feature is represented as a decision node. Depending on the value of this feature, the tree branches into two leaves, each containing a specific value that is added to the model’s output. In Figure 1 we show the creation of a decision tree where the node is represented by a SNP.

XGBoost’s feature importance scores are used to rank the most influential features contributing to the prediction. The ability to quantify feature importance is one of XGBoost’s strengths, allowing us to interpret which features have the most significant impact on the model’s predictions. This capability is often used in feature selection before training the real model.

XGBoost can be used to train boosted trees, random forests of decision trees, or random forests of boosted trees.

We used XGBoost to develop a model of boosted trees where each tree is trained to correct the mistakes made by previous trees. A tree is built by iteratively splitting a leaf formed by a set of data points.

The gain of a feature f is used to split the leaf into two leaves, picking the f that has maximum gain (Equation 1). It is defined as (Chen and Guestrin, 2016):

$${\text{gain}}_{f}=\frac{1}{2}\left[\frac{G_{L}^{2}}{H_{L}+\lambda }+\frac{G_{R}^{2}}{H_{R}+\lambda }-\frac{{\left(G_{L}+G_{R}\right)}^{2}}{H_{L}+H_{R}+\lambda }\right]-\gamma$$ (1)

where the first two fractions are the contribution of the left and right leaves, and the third is the contribution of the original leaf. The parameters λ and γ are L1 and L2 regularization terms used for pruning: if the gain is negative, the leaf is not split.

Beyond the construction of decision trees, the gain can be also used as a score to get insights into the significance of each feature. The actual model is trained using the plain genotype coded as an integer vector (see Section 3.1) without any additional preprocessing.

#### 2.1.1 Training parameters

The hyperparameters used for the model were: the number of trees (ranged from 10 to 100); the grow policy (either loss-guide or depth-wise); the learning rate (ranged from 0.01 to 0.2); the maximum tree depth (between four and 6); the minimum child weight (between one and 3); λ (ranged from 0 to 5); and γ (ranged from 0 to 5) that can be tuned to add complexity or limit overfitting. The best hyperparameters are chosen with the help of a grid-search.

### 2.2 COMBI

The two methods that carry the name COMBI aim at examining the relation between SNPs and phenotypical traits (Mieth et al., 2016) and represent the basis of the interpretable machine learning paradigms in bioinformatics for the analysis of human DNA. These paradigms focus on the explainability of certain traits while still offering predictive capability, and aim at maximizing both of these aspects of classification. To this end, COMBI uses a support vector machine model (Cortes and Vapnik, 1995) applied to the Wellcome Trust Case Control Consortium (WTCCC) data of human genome-disease association (Jones et al., 2007), taking advantage of the direct mathematical correlation it provides between inputs and outputs.

The decision-making process of machine learning algorithms is usually black-box, limiting the interpretability of results in complex contexts such as SNP data and other biological data. COMBI has proved extremely useful in providing an answer to this problem, like detecting genetic risk scores for quantifying patients’ predisposition to disease on the WTCCC (Marigorta et al., 2018), advancing precision medicine in the field of oncology for therapy targeted to each patient (Asada et al., 2021), and predicting susceptibility to asthma based on SNP information of individuals (Gaudillo et al., 2019).

Recently, deep learning has emerged as a powerful classification tool, with the marked need to make its results interpretable and robust for appropriate use in more general contexts (Zou et al., 2019). To achieve this goal, research is moving towards machine learning explainability in genomics (Watson, 2022). All these factors led to the emergence of DeepCOMBI (Mieth et al., 2021), a neural network-based classifier that uses layer relevance propagation (Samek et al., 2016) to achieve the same level of explainability as the original COMBI model, with increased performance on the same WTCCC dataset. DeepCOMBI has successfully been applied to the study of the response of rheumatoid arthritis patients to certain medication based on their genome data, helping to better identify non-responders (Lim et al., 2022), and for improving risk prediction of developing schizophrenia, a highly inheritable disorder whose genetic markers are still unclear (Martins et al., 2024).

In this study, the COMBI framework consists of the testing and adaptation of the methods used by COMBI (Mieth et al., 2016) with the Support Vector Machine (SVM) model and DeepCOMBI (Mieth et al., 2021) with the Multilayer Perceptron (MLP) model.

#### 2.2.1 COMBI support vector machine model

For the SVM model, a linear kernel was chosen to guarantee a direct correlation between the weight of each input SNP feature and the final prediction. This way the predictions of the model can be encoded with the simple expression (Equation 2) as (Cortes and Vapnik, 1995):

$$Y=W^{T}X+b$$ (2)

where X is the vertical stack of feature vectors of each individual, and the weight matrix W is the vertical stack of weights given to each feature. Higher weight features will have more relevance in deciding the final result.

The SVM tries to find the best separating hyperplane between the two sets of data and takes in input every SNP feature of each individual, one-hot encoded as {1,0,0} if both alleles are the same as the reference genome, {0,1,0} if one allele is different, and {0,0,1} if both alleles are different.

#### 2.2.2 DeepCOMBI multilayer perceptron model

**FIGURE 2:**
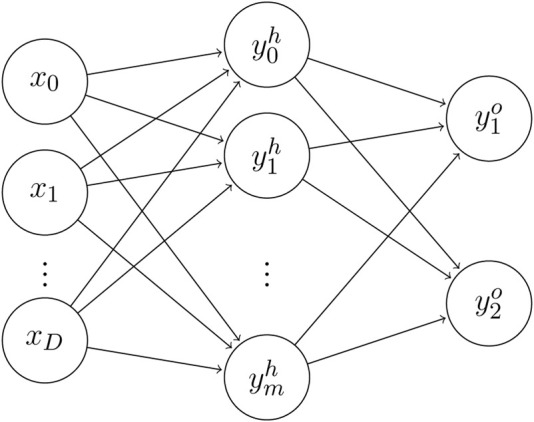
Network graph of a single hidden layer perceptron with D input units and two output units. The parameters of our networks are: D=3×n , where n is the length of the sequence (due to one-hot encoding on a sequence of SNP), and h=128 .

The MLP model was constructed with an input layer, a hidden layer and an output layer, as shown in Figure 2. Due to the high dimensionality of the SNP features treated in this study, much more complex models would require too much time to train. The model is composed of an input layer with as many neurons as three times the number of SNPs, due to the same one-hot encoding of the data as seen in the SVM. The size of the hidden layer is a modifiable hyperparameter of the MLP, while the output layer is composed of two neurons corresponding to the two phenotypes (alive/dead) in the dataset.

The model learns to distinguish between the two phenotypes by using a forward pass of the data to make decisions, and a backward pass to update its parameters based on the correctness of the forward pass. Every epoch a forward and backward pass of all the data is performed. Each neuron stores a value that depends on the previous layer of neurons as such (Equation 3):

$$y_{n}^{l+1}=F\left(\underset{i\in l}{\sum }w_{i,n}\cdot y_{i}^{l}+b_{i,n}\right)$$ (3)

where $w_{i,n}$ and $b_{i,n}$ are the weight parameter and bias between neuron i of layer l , and neuron n of layer l+1 and F is the activation function used to add non-linearity to the model. The backward pass updates the weights $w_{i,n}$ so that the model better falls in line with the previously processed data’s true labels, using an algorithm known as stochastic gradient descent.

#### 2.2.3 Training parameters

The SVM model was trained until convergence and its optimal hyper-parameters were found through a grid-search procedure. The hyper-parameters used for the SVM model were: L2 regularization with C = 100(1, 100) and squared hinge loss (Lee and Lin, 2013).

The hyperparameters used for the MLP model were optimized via grid search. The number of hidden neurons varied in the range 128–256-512; the optimal value was 128, which provided the best trade-off between model capacity and generalization performance. The dropout rate was varied between 0.0 and 0.5 (0.1 steps), with an optimal value of 0.3. ReLU activation function was used. L1 and L2 regularization weights were explored in the range 0.0001–0.1, with optimal values of 0.1 and 0.01 respectively, as larger regularization strengths were found beneficial to mitigate overfitting. The learning rate was set at $10^{-12}\;\left(10^{-14},10^{-3}\right)$ with binary cross-entropy loss. A multiplying factor of 10 was used for each step. The number of training epochs varied between 100 and 1000; the optimal value of 1000 epochs ensured convergence without further gains beyond this threshold.

### 2.3 Chaos Game Representation

Chaos Game Representation (CGR) is an iterative mapping technique to transforms a sequence defined over an alphabet Σ into an image. In CGR a sequence is represented as a unique pattern and it is mapped to unique coordinates. For any sequence, regardless of its length and background, CGR can encode it into an image by representing each feature through a point identified by coordinates. Furthermore, by knowing the coordinates of a feature, CGR allows one to inference the input sequence.

The application of CGR in bioinformatics was first proposed in (Jeffrey, 1990), where an encoding scheme for genomic sequences into squares was first proposed. In this representation each vertex of the square corresponds to one of the four DNA nucleotides, with Σ={A,C,G,T} . Extension to the framework have followed, involving also RNA, proteins and physio-chemical properties (Kania and Sarapata, 2022; Dick and Green, 2020; Akbari Rokn Abadi et al., 2023).

In bioinformatics CGR has been used in alignment-free sequence comparison to reconstruct phylogeny trees (Almeida et al., 2001; Lichtblau, 2019; Kania and Sarapata, 2021) and as a feature encoding for machine learning and deep learning approaches (Löchel and Heider, 2021; Avila Cartes et al., 2022).

CGR was initially proposed to overcome limitations of traditional sequence analysis by offering an alignment-free, visually intuitive method to study DNA sequences. Its main strength lies in its ability to implicitly encoding information about the patterns distribution of the sequence, revealing hidden structures and genomic signatures. CGR algorithm has a low memory footprint, a complexity that is linear in the input sequence and it is adaptable to modern data analysis techniques, making it an attractive alternative to other methods. Moreover, the exceptional classification capability of Neural Networks in image recognition have started a new wave of interest in CGR in bioinformatics. While genomic and proteomic applications have been subject of much attention, to the best of our knowledge, the CGR encoding of sequences of consecutive SNPs has not been investigated yet, thus raising our interest in testing this approach.

#### 2.3.1 Chaos game representation algorithm

The Chaos Game Representation algorithm transforms genomic sequences into images.

Let $s=s_{1}\ldots s_{n}$ be a sequence defined over the alphabet Σ={A,C,G,T} . Then the CGR encoding of the sequence s is the bidimensional representation of the ordered set of pairs $\left\{\left(x_{i},y_{i}\right),\;0\leq i\leq n\right\}$ , where the pair $\left(x_{i},y_{i}\right)$ is iteratively defined (Equations 4, 5) as (Avila Cartes et al., 2022; Löchel and Heider, 2021):

$$\left(x_{i},y_{i}\right)=\frac{1}{2}\left(\left(x_{i-1},y_{i-1}\right)+g\left(s_{i}\right)\right)\;with\;i\geq 1$$ (4)

where the origin $O\left(x_{0},y_{0}\right)=\left(0,0\right)$ and

$$g\left(s_{i}\right)=\left\{\begin{array}{c} \left(-1,1\right)\;s_{i}=A\\ \left(-1,-1\right)s_{i}=C\\ \left(1,1\right)s_{i}=T\\ \left(1,-1\right)\;s_{i}=G \end{array}\right.$$ (5)

We have extended this definition implementing a Chaos Game Representation model that consists of an encoder unit that converts a genotype sequence into a CGR image and a classifier unit that executes the classification.

#### 2.3.2 Encoder unit

**FIGURE 3:**
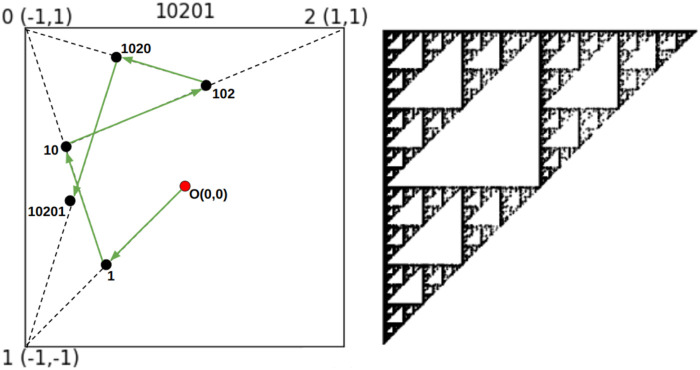
The Chaos Game Representation applied to genotype sequence. The graphical-conceptual application of CGR for genotype (left) and image conversion for a sea bass with Active10 SNP features (right).

To apply the Chaos Game Representation algorithm to the context of sea bass genetic data, we modified its genomic application to genotype sequences. Our proposed CGR encoding for genotype keeps the square representation assigning a genotype to each of the vertices except one, for backward compatibility with genomic sequences, and maintains the distribution of genotype s within the image clear (Figure 3).

Let $h=h_{1}\ldots h_{n}$ be a sequence defined over the alphabet Σ={0,1,2} . Then the CGR encoding of the sequence h is the bidimensional representation of the ordered set of pairs $\left\{\left(x_{i},y_{i}\right),\;0\leq i\leq n\right\}$ , where the pair $\left(x_{i},y_{i}\right)$ is iteratively defined (Equations 6, 7) as:

$$\left(x_{i},y_{i}\right)=\frac{1}{2}\left(\left(x_{i-1},y_{i-1}\right)+g\left(h_{i}\right)\right)\;with\;i\geq 1$$ (6)

where the origin $O\left(x_{0},y_{0}\right)=\left(0,0\right)$ and

$$g\left(h_{i}\right)=\left\{\begin{array}{c} \left(-1,1\right)\;h_{i}=0\\ \left(-1,-1\right)h_{i}=1\\ \left(1,1\right)h_{i}=2 \end{array}\right.$$ (7)

Note that any fixed bijection g(⋅) would work, as the encoded information depends on the sequence itself. For example, if g(0)=(1,−1) we would have obtained a symmetric image, with respect to the diagonal, to the one shown in Figure 3.

The sequence h is obtained by sorting the SNPs according to their physical position in the reference genome, following the order of the contigs/scaffolds provided by the assembly and, within each contig, according to increasing genomic coordinates (in bp).

#### 2.3.3 Classifier unit

Our classifier unit consists of a Deep Convolutional Neural Network (DCNN). We chose to use DCNN due to the large number of SNP features selected against the small number of sea basses in the analyzed datasets. We instantiated and tested several networks: AlexNet (Krizhevsky et al., 2017), ResNet50 and ResNet101 (He et al., 2016).

AlexNet and ResNet are three-channel architectures and require a fixed image size for training; 227×227 for AlexNet and 224×224 for ResNet. We replicated the content of the single channel in each of the three channels and resized the images, with dimensions consistent with networks, using bicubic interpolation (Lundh et al., 2024). All CNN backbones (AlexNet, ResNet50, and ResNet101) were fine-tuned end-to-end for the CGR-based classification task. The final fully connected layer of each network was replaced with a single-neuron layer with sigmoid activation, suitable for binary classification.

#### 2.3.4 Training parameters

The hyper-parameters used for the model were the last dense layer with one neuron for the binary classification; SGD optimizer with learning rate $10^{-3}$ and the binary cross-entropy loss function; 5-fold cross-validation of training on the data.

A series of experiments was conducted by varying the number of epochs and batch size to identify the configuration yielding the best validation performance. The number of epochs ranged from 30 to 120, and batch sizes varied from 15 to 30, due to the high dimensionality of the SNP features and the low-rank data treated in this study. To enhance reproducibility, we report the final training parameters used for each network architecture:

- AlexNet: batch size = 15, epochs = 120;
- ResNet50: batch size = 30, epochs = 90;
- ResNet101: batch size = 15, epochs = 30.

Further details regarding network architectures, input pre-processing, and final training parameters are provided in Supplementary Material.

## 3 Results and discussion

This section presents the aquaculture data we used in our research, the experimental setup instantiated on our classification models, and the phenotype classification results we obtained on each model and experiment performed.

### 3.1 Datasets

Data used in our research refer to the study of (Mukiibi et al., 2025). In that study, 990 juvenile European sea bass (6–20 g), produced following a full-factorial mating scheme using 25 sires and 25 dam, were subjected to a 29 days VNN challenge test. Mortality was individually recorded as a binary trait (alive or dead at the end of the challenge trial). Phenotypes were fairly balanced having 54.24% of alive fish at the end of the challenge test and 45.76% of dead fish. Sires, dams and 40 offspring were whole-genome sequenced, whereas the remaining offspring were genotyped using the MedFish SNP array Peñaloza et al. (2021) consisting of about 30K SNPs. These animals were then imputed to whole-genome sequence, obtaining a high-dimensional genotype data consisting of 6,072,853 SNPs for each fish. Since European sea bass is a diploid organism, it has two alleles at the SNP and two possible variations from the reference allele. Genomic data included:

- The SNP feature identifier, composed by chromosome number and location of each SNP in base pairs;
- The number of copies of the reference allele (in this case, the minor allele), that can be 0, one or 2.

Having only 990 samples, the feature matrix is low-rank, making it exceptionally difficult to analyze. To overcome the data’s high dimensionality, we will use three groups of features selected on the basis of functional genomic information: 1) Tissue specific, 2) Active, and 3) Control. Tissue-specific refers to genetic variants located in open chromatin regions based on ATAC-seq data obtained from two key tissues in VNN, brain and head kidney, sampling 10 fish either after infection or mock-infected. Active refers to active regulatory regions based on ATAC- and ChIP-seq data from several different tissue types (brain, gill, liver, gonads, skeletal muscle and head kidney) in control fish (Mukiibi et al., 2025). Active datasets consist of SNPs included in regulatory regions that were found to be active across at least 80%, 50% and 10% of analyzed tissues. Control datasets, numerically proportional to the Active ones, contain randomly selected SNP features that were located within non-active regions (i.e., quiescent regions).

Tissue-specific datasets are very large datasets with a large number of SNPs (about a million); instead, Active and Control datasets have a far fewer number of SNPs (thousands or tens of thousands), further details are reported in Table 1.

**TABLE 1:** Information on the composition of the datasets in Tissue-specific, Active and Control categories.

Category	Dataset	# SNPs
Tissue-specific	Hk_NNV	1,193,048
	Hk_mock	1,082,100
	Br_NNV	775,840
	Br_mock	832,801
Active	Active80	6,862
	Active50	11,130
	Active10	80,768
Control	Control80	6,862
	Control50	11,130
	Control10	80,768

Randomly partitioning data into k equally sized subsets for k -fold cross-validation can lead to overly optimistic results, particularly when the dataset includes closely related individuals from a single generation, resulting in many sibs being present in both the training and testing sets (Clark et al., 2012; Pérez-Cabal et al., 2012; Faggion et al., 2022; Fraslin et al., 2022; Faggion et al., 2023). In realistic selective breeding scenarios, the genomic relationships between training and testing sets, such as those resulting from half- and full-sib family structures, progressively weaken across successive generations, leading to a gradual disruption of family-based linkage disequilibrium (de los Campos et al., 2013). To mimic this scenario, a strategy based on K-means method was adopted to cluster the animals according to distance of genomic relationships (Yang et al., 2010). Using genomic information, two clusters (cluster 0 and cluster 1) were then created reducing the relatedness between clusters while maximizing the within-cluster relatedness. K-means clustering was performed using the function kmeans (stats/R) which implements the Hartigan and Wong algorithm (Hartigan and Wong, 2018). Number of animals in each cluster and the classification by phenotypes is shown in Table 2, whereas the average genomic relatedness (SD) within cluster and the average genomic relatedness (SD) between clusters are reported in Table 3. As previously explained, the experimental animals were produced through a full-factorial mating scheme using breeders from the same commercial population. For this reason, the average genomic relatedness between clusters (−0.045) is not as low as would be expected if the animals belonged to different populations or were characterized by distinct genetic backgrounds. Nevertheless, this approach provided the most suitable strategy to make the procedure sufficiently challenging given the available data.

**TABLE 2:** Distribution of sea bass by genomically distant clusters and phenotypes.

Clusters	# sea basses	Phenotypes	
		Alive	Dead
Cluster 0	589	373	216
Cluster 1	401	164	237
Total	990	537	453

**TABLE 3:** Average genomic relatedness (SD) within each cluster (on diagonal) and average genomic relatedness (SD) between clusters (off diagonal).

Clusters	Cluster 0	Cluster 1
Cluster 0	0.029 (0.077)	−0.045 (0.062)
Cluster 1		0.064 (0.097)

### 3.2 Experimental setup

Given the high-dimensional low sample size nature of the dataset, specific strategies were adopted to ensure stable model training and to mitigate overfitting. First, dimensionality was reduced through biologically informed SNP selection, focusing on variants located in functionally active genomic regions identified via ATAC-seq and ChIP-seq data, thereby improving the signal-to-noise ratio.

Second, the selected machine learning models incorporate mechanisms that are well-suited for high-dimensional data. In particular, XGBoost employs regularization and tree-based feature selection, while the COMBI SVM framework relies on linear kernels and embedded feature selection to handle large feature spaces efficiently. Deep learning approaches (DeepCOMBI and CGR-based CNN) were designed with controlled model complexity and trained using cross-validation to reduce the risk of overfitting.

Finally, model evaluation was performed using both standard random splits and more stringent genomically distant partitions, ensuring that the models are assessed under conditions that reflect realistic generalization in the presence of high-dimensional genomic variability.

We carried out several tests on a random partition and on the two genomically distant clusters. In all experiments, data partitioning was designed to ensure a clear separation between the training, validation, and testing phases. In particular, an independent test set was always defined, which was never used during the model training or validation phases. The remaining data was used for training, including an internal validation phase obtained through stratified cross-validation. In the case of random partitioning, the dataset was divided into 80% used for training and validation and 20% reserved for independent testing.

In experiments on genomically distant clusters, the models were trained on the larger cluster (cluster 0), while the other cluster (cluster 1) was used as an independent test set. In both cases, the training phase included 5-fold cross-validation, in which approximately 4/5 of the data in each fold was used for model training and 1/5 for validation, used to monitor performance during training.

In the case of random partitioning, we adopted an 80:20 train-test split as a standard baseline evaluation strategy widely used in machine learning. This choice provides a balance between ensuring sufficient data for model training while preserving an independent test set for unbiased performance evaluation.

However, we acknowledge that in high-dimensional low sample size settings, such splits may lead to optimistic performance estimates. For this reason, we complemented the 80:20 evaluation with a more stringent and biologically meaningful validation strategy based on genomically distant population splits, in which training and test sets consist of genetically dissimilar individuals. This second evaluation provides a more realistic assessment of model generalization in practical breeding scenarios.

A limitation of this study is the lack of validation on an independent external dataset, due to the limited availability of comparable genomic resources for VNN resistance in European sea bass. To mitigate this, we adopted a validation strategy based on genomically distant population splits, which provides a more realistic assessment of model generalization by reducing the influence of genetic relatedness between training and test samples. This approach can be considered a practical proxy for external validation in aquaculture breeding contexts.

Given the high dimensionality of the data, we used the features selected based on biological significance. We considered first the Tissue-specific datasets, which are more extensive. Then we move to the Active datasets that contain far fewer features compared to the Tissue-specific ones, helping to assess whether the number of SNPs influences classification performance. To assess the effectiveness of the selected SNPs for classification, we compared the models trained on the Active selections with Control models using random SNP selections of the same size. These Control datasets allowed us to verify whether the SNPs chosen based on biological relevance were genuinely contributing to improved classification performance.

To further assess model training behavior, we report representative training metrics for the CGR-based deep learning approach (see Supplementary Table S2). These values, extracted from experimental logs, show consistent convergence patterns, with stable loss values and validation accuracy aligned with the overall classification performance.

For the other models considered in this study (XGBoost, COMBI SVM, and DeepCOMBI), overfitting was controlled through algorithm-specific mechanisms such as regularization, feature selection, and cross-validation. In particular, these models do not rely on iterative epoch-based optimization in the same way as deep convolutional networks, making loss curves less informative. Instead, their generalization ability was assessed through stratified cross-validation and evaluation on independent test sets, including the more stringent genomically distant data partitions.

Four machine learning models were employed: XGBoost, COMBI SVM, DeepCOMBI, and CGR encoding with a CNN classifier.

The hyperparameters for each model were set through grid search as specified in Section 2 Materials and Methods. They are the optimal values within the explored ranges. The models were evaluated using accuracy, precision, recall, and F1-score.

### 3.3 Classification performance analysis

In this section we report the results obtained by each model, tested on the various splits of data described above. The main observation to highlight for these tests is that the task at hand is quite challenging, as we know the mortality phenotype taken in exam is not exclusively determined by the genotype, so all results reflect this difficulty. This section will only list the F1-score as a performance metric for space limitations, but the complete set of results can be found in the Supplementary Material.

#### 3.3.1 Random partition tests

The first benchmark used was the random 80–20 partition for the individuals in the Tissue-specific datasets, as is standard practice in many machine learning applications and tasks. Tests on this split are a useful metric to compare the efficacy of these approaches with regard to the rest of the literature on the subject, minding the challenge of the data at hand. The results of these tests are reported in Table 4.

**TABLE 4:** F1-scores obtained on the Tissue-specific tests using the random partition split. COMBI SVM failed on head-kidney data because the maximum allowed variable size in the underlying LIBLINEAR (Fan et al., 2008) library is exceeded by the amount of data in these datasets. As a workaround, we run COMBI-SVM on each chromosome and reported the average F1 (data in parenthesis).

Dataset	XGBoost	COMBI SVM	Deep-COMBI	CGR
Hk_NNV	0.53	N/A (0.52)	0.53	0.15
Hk_mock	0.53	N/A (0.48)	0.61	0.58
Br_NNV	0.58	0.62	0.61	0.58
Br_mock	0.58	0.62	0.48	0.26

Note that the COMBI SVM method uses the underlying LIBLINEAR (Fan et al., 2008) software library for its implementation, which has a maximum allowed variable size that is exceeded by the amount of data in the head-kidney tissues. As a workaround, we run COMBI-SVM on each chromosome and reported the average F1.

The best performance we were able to achieve on the random partition tests is using the COMBI SVM approach on the sets that allowed it, reaching 62% F1-score, while the head-kidney datasets have proven more challenging for XGBoost. Both of the neural network-based DeepCOMBI and CGR methods encounter significantly more difficulty in the Hk_NNV and Br_mock datasets, but achieve 61% and 58% F1-score respectively on Hk_mock and Br_NNV. As an additional test (Supplementary Material) we have studied the correlation between the predictions of XGBoost and Deep-COMBI, but none was observed as significant.

The head-kidney and brain tissue data contain an extremely large number of SNPs, since all four data sets represent accessible, but not necessarily active genomic regions. As a means to significantly reduce the number of features, we decided to use more detailed functional information, including ChIP/seq data. This allowed to identify active regulatory elements. Despite the inclusion of a larger number of tissues, focusing on active regions only enabled a drastic reduction of features, while preserving core information on biological importance.

This way, three datasets containing Active SNPs were tested. To ascertain the quality of these selections, randomly sampled datasets of the same size were used as Control sets, with the expectation that the Active SNP selections would yield higher performance than the Control sets because of their careful filtering process. The comparison of the above tests using the same random fixed 80%–20% split as above can be found in Table 5.

**TABLE 5:** F1-scores obtained on the Active and Control tests using the random partition split.

Dataset	XGBoost	COMBI SVM	Deep-COMBI	CGR
Active80	0.60	0.62	0.42	0.58
Control80	0.51	0.63	0.00	0.10
Active50	0.57	0.60	0.62	0.48
Control50	0.57	0.61	0.04	0.40
Active10	0.58	0.62	0.53	0.52
Control10	0.61	0.63	0.39	0.00

The tests using Active and Control subsets reveal to which extent each model is able to distinguish between high-quality SNP data and random noise. XGBoost seems to distinguish well between Active80 and Control80, but loses this ability on the 50% and 10% variants, while COMBI SVM seems to be unable to make meaningful distinctions between any couple of Active and Control sets. As a confirmation, the correlation between the predictions obtained using the Active datasetes and the predictions obtained using the Control datasets were significant when using XGBoost or COMBI-SVM (Supplementary Material). On the other end, both neural network-based approaches display marked gaps in F1-score between Active and Control sets, often with many decimal points of difference. This would suggest that these methods are better suited at distinguishing the random noise of the control sets from more meaningful SNP data.

The tools examined in this study also provide the opportunity to investigate the relevance of features or SNPs used during the classification process. For instance, in XGBoost, each feature contributing to the classification is associated with a gain value, which can be further analyzed to assess its importance. Similarly, in COMBI SVM, the contribution of each chromosome to the classification can be evaluated using chromosome-specific AUC values. An example of this type of analysis is shown in Supplementary Table S2 (Supplementary Material).

For both XGBoost and COMBI SVM, we report chromosome-level gain or AUC values obtained from the random partition test. Notably, for both methods, chromosome three emerges as the most influential for classification. At a finer level of detail, XGBoost also allows us to identify the top 10 SNPs ranked by gain, as reported in Supplementary Table S3 (Supplementary Material). Across both tables, chromosome three consistently appears as the most relevant, with particular emphasis on the SNP located at position 10077301, which has been associated with resistance to Viral Nervous Necrosis (VNN). This SNP corresponds to a major quantitative trait locus (QTL) for VNN resistance, as recently reported in (Mukiibi et al., 2025).

Although this analysis is preliminary, it highlights the potential to link feature importance measures to specific genomic regions. Such connections can support biological validation and help bridge the gap between predictive performance and biological interpretation.

#### 3.3.2 Genomically distant clusters tests

The results of the tests performed using the partition of genomically distant individuals in clusters are summarized in Table 6. The clusters were selected to be as genomically distant from each other as possible, making the expectation for this task to show worse overall performance than those of the random partition tests. In these tests, the COMBI SVM framework was able to process all data due to the smaller training sets.

**TABLE 6:** F1-scores obtained on the Tissue-specific tests using the genomically distant split.

Dataset	XGBoost	COMBI SVM	Deep-COMBI	CGR
Hk_NNV	0.60	0.41	0.09	0.40
Hk_mock	0.49	0.41	0.34	0.00
Br_NNV	0.59	0.42	0.71	0.31
Br_mock	0.51	0.41	0.46	0.67

On these data splits we can see how, as expected, the difficulty of the problem notably increases, due to the much smaller training set size and groups specifically selected in a way to contain individuals as genomically different as possible, making the prediction of the phenotype overall much harder. In spite of this, some results go even beyond what the 80%–20% tests were able to achieve, with markedly high F1-scores of 60% on Hk_NNV by XGBoost, 71% on Br_NNV by DeepCOMBI, and 67% on Br_mock by CGR. The Hk_mock dataset becomes very challenging for all methods, and COMBI SVM performs badly on all Tissue-specific datasets.

Lastly, the same genomically distant test was performed as before on the Active and Control datasets, listed in Table 7. Using these clusters, the Active and Control tests also show more ambiguous results than before. On the 80% sets, all methods except COMBI SVM struggle to distinguish between the random and meaningful data. We believe this behavior might depend on the relatively small number of SNPs that are included in these datasets. The 50% sets show good discriminatory capabilities on all models, with gaps of many decimal points between Active and Control. Active10 and Control10 are interestingly only a few decimal points apart on every test, but with Active10 always in the lead, giving the impression that there is just enough difference to meaningfully distinguish the two. When it comes to overall classification performance, the CGR model outperforms all the other ones on the Active sets, often nearing or exceeding 70% F1-score, suggesting that, despite the diversity of the clusters, there are features highlighted by the CGR representation that can meaningfully distinguish between the two phenotypes. When measuring the correlation between the predictions with an Active dataset and the predictions with the corresponding Control dataset (Supplementary Material), only SVM showed a significant correlation, confirming that its predictions are not affected by the choice of the SNPs.

**TABLE 7:** F1-scores obtained on the active and control tests using the genomically distant split.

Dataset	XG-Boost	COMBI SVM	Deep-COMBI	CGR
Active80	0.58	0.50	0.25	0.62
Control80	0.63	0.43	0.44	0.74
Active50	0.66	0.49	0.70	0.74
Control50	0.62	0.43	0.42	0.55
Active10	0.64	0.42	0.72	0.76
Control10	0.61	0.39	0.68	0.74

In summary, XGBoost does not often perform the best, but among all models it is the one that most consistently obtained reliable results often reaching around 60% F1-score on all the above tests. We believe that the stable performance observed for XGBoost can largely be attributed to its built-in regularization and anti-overfitting mechanisms. In particular, the explicit regularization terms on tree complexity, together with shrinkage (learning rate), row and column subsampling, help control model variance and prevent overfitting. These properties are especially beneficial in high-dimensional settings with relatively small sample sizes. As a result, XGBoost tends to generalize more robustly across different experimental configurations, which is consistent with the behavior observed in our results. COMBI SVM reaches a similar level of reliability on the 80%–20% split tests, but it finds significantly more difficulty in classification between the two clusters. This loss of performance for SVM is observed specifically in the experiments with the two distant populations, across all datasets. This suggests that there could be some structural difference in the populations that negatively impacts on the results when a SVM is used. Another, possibly additional, reason could be that the training set, besides being composed by individuals with the largest genetic distance with respect to the test, is also smaller than the one used in the 80-20 test. It is therefore possible that also due to the large number of ternary features of the one-hot encoding used by the SVM, and the smaller number of training examples that could affect generalization, even small differences lead to a different, possibly wrong, classification with the linear hyperplane separation used by the SVM.

DeepCOMBI’s performance is often inconsistent, ranging from very good F1-score around 70%, to extremely poor under 10%. CGR is similarly inconsistent in most cases, with high peaks and low valleys, but emerges when used for the genomic distant population tests on the split with SNPs active across at least 50% of the tissues. The good performance of CGR in this specific scenario can be explained by the characteristics of the data. Although Active datasets are relatively small compared to Tissue-specific datasets, they contain SNPs in active regulatory regions, presenting a clearer biological signal and less noise. This allows the model to learn concrete patterns from CGR images without being distracted by noise. In this context, CGR representation, which simultaneously preserves the order and position of SNPs along the sequence, could facilitate the emergence of global spatial patterns rather than relying on individual, highly population-specific variants. It is reasonable to think that the CGR image is not “recognizing only individual SNPs”, but rather the way in which functional SNPs are arranged along the genome, and that this pattern remains unchanged even between genetically diverse populations.

However, even for CGR, just focusing on Active regions might not be enough. Our results show that the best performances are obtained with SNPs shared by at least 50% of the tissues. If we lessen the condition, requiring the sharing by at least 10% of the tissues, then lot of noise also flows in, and the model become unable to distinguish between active and control. On the other end, if we restrict the condition, imposing the sharing among 80% of the tissues, then the sequences are too few and again the model fails to distinguish among the two classes.

In conclusion, in our experiments, CGR encoding showed a better ability to captured spatial patterns relevant for prediction even in genomically distant populations when the SNPS are shared by at least 50% of the tissues, but it also showed to be sensitive to overly noisy datasets, such as Tissue-specific ones.

As previously explained in Subsection 3.1, the data were collected from animals belonging to a single commercial population; the limited availability of data is largely due to the experimental procedures required to assess disease-resistance traits, in addition to the complexity of sequencing and functional annotation analyses. Therefore, the research presented should be considered a case study, and the results should be interpreted within this context. Future investigations are required, particularly to validate these methods across multiple sea bass populations, as well as to extend their application to other species and to different oligogenic or polygenic traits of economic importance to the aquaculture sector.

Because the genetic architecture of complex traits is largely influenced by non-coding variants located within regulatory regions, this study introduces an innovative strategy that prioritizes genetic variants with the strongest effects on the phenotype under investigation, based on ATAC- and ChIP-seq analyses. The comparison between models incorporating SNPs located within active regulatory regions and models including randomly selected SNPs from quiescent regions highlights, in almost all cases, the potential benefits of integrating functional information into classification models. Clustering individuals based on genomic relationships represented the most challenging scenario achievable with the available data, mimicking long-term selection conditions while minimizing the risk of inflated accuracy estimates arising from the presence of half- and full-sibs in both training and testing sets. The results indicate that, when integrated with machine learning approaches, this strategy has the potential to be effective in real-world selective breeding scenarios, particularly when candidate breeders are evaluated using data from a reference population that does not include close relatives of the animals under selection.

### 3.4 Time and memory usage

This section reports the amount of time and memory used for training by each of the methods on the 80%–20% (Tables 8, 9) and on the genomically distant data splits (Tables 10, 11). The time for testing was negligible for all the tools in comparison to the time for training.

**TABLE 8:** Training time on the 80–20 partition for all tools. The CGR column reports the total time, including the time for image encoding that could be the result of a pre-processing. In parenthesis we report the actual time for the training task.

Dataset	# SNPs	XGBoost	COMBI SVM	DeepCOMBI	Chaos game representation
Hk_NNV	1,193,048	20 m 42s	09 h 28 m 22s	126 h 49 m 56s	22 h 19 m 16s (10 m 41s)
Hk_mock	1,082,100	19 m 24s	08 h 28 m 14s	117 h 30 m 35s	15 h 03 m 09s (07 m 23s)
Br_NNV	775,840	16 m 19s	06 h 54 m 12s	87 h 29 m 24s	05 h 28 m 32s (07 m 31s)
Br_mock	832,801	16 m 39s	06 h 51 m 15s	90 h 33 m 40s	07 h 04 m 04s (07 m 24s)
Active10	80,768	10 m 06s	01 h 01 m 29s	07 h 44 m 45s	31 m 06s (17 m 19s)
Control10	80,768	08 m 26s	01 h 08 m 16s	06 h 58 m 35s	39 m 43s (27 m 05s)
Active50	11,130	09 m 01s	30 m 15s	01 h 12 m 54s	10 m 45s (07 m 56s)
Control50	11,130	08 m 51s	29 m 47s	01 h 57 m 25s	25 m 28s (22 m 08s)
Active80	6,862	08 m 12s	21 m 13s	01 h 18 m 02s	12 m 19s (10 m 27s)
Control80	6,862	08 m 13s	28 m 35s	01 h 25 m 58s	14 m 28s (11 m 39s)

**TABLE 9:** Memory usage (in GB) for the 80–20 partition for all tools. The CGR column reports both the peak of memory for image encoding and the peak of memory for the training task.

Dataset	# SNPs	XGBoost	COMBI SVM	DeepCOMBI	Chaos game representation
Hk_NNV	1,193,048	143,13	149.53	147.60	28.85–12.76
Hk_mock	1,082,100	143.67	149.60	147.96	24.70–12.88
Br_NNV	775,840	146.92	149.68	148.18	31.30–18.73
Br_mock	832,801	143.37	149.01	149.26	17.11–12.89
Active10	80,768	147.22	149.79	149.18	2.04–15.09
Control10	80,768	145.27	148.68	146.69	2.15–15.14
Active50	11,130	144.77	147.51	149.19	2.01–15.14
Control50	11,130	145.62	148.94	148.99	2.06–15.13
Active80	6,862	145.22	148.51	148.04	2.48–12.89
Control80	6,892	145.34	147.70	148.15	2.06–15.11

**TABLE 10:** Training time on the divergent partition for all tools. The CGR column reports the total time, including the time for image encoding that could be the result of a pre-processing. In parenthesis we report the actual time for the training task.

Dataset	# SNPs	XGBoost	COMBI SVM	DeepCOMBI	Chaos game representation
Hk_NNV	1,193,048	19 m 26s	08 h 29 m 50s	151 h 55 m 57s	22 h 22 m 40s (14 m 05s)
Hk_mock	1,082,100	18 m 14s	07 h 49 m 32s	136 h 28 m 25s	15 h 02 m 33s (06 m 47s)
Br_NNV	775,840	15 m 04s	06 h 12 m 09s	65 h 35 m 58s	05 h 34 m 22s (13 m 21s)
Br_mock	832,801	16 m 51s	06 h 10 m 16s	76 h 20 m 04s	07 h 03 m 27s (06 m 47s)
Active10	80,768	09 m 07s	01 h 02 m 13s	08 h 45 m 26s	25 m 39s (11 m 52s)
Control10	80,768	08 m 11s	01 h 05 m 02s	05 h 35 m 22s	25 m 39s (11 m 59s)
Active50	11,130	08 m 36s	23 m 26s	01 h 01 m 41s	15 m 00s (12 m 11s)
Control50	11,130	08 m 37s	34 m 34s	57 m 10s	15 m 55s (12 m 35s)
Active80	6,862	09 m 04s	30 m 52s	42 m 11s	19 m 28s (17 m 36s)
Control80	6,892	08 m 24s	27 m 44s	44 m 28s	14 m 47s (11 m 58s)

**TABLE 11:** Memory usage (in GB) for the divergent partition for all tools. The CGR column reports both the peak of memory for image encoding and the peak of memory for the training task.

Dataset	# SNPs	XGBoost	COMBI SVM	DeepCOMBI	Chaos game representation
Hk_NNV	1,193,048	142.87	149.29	148.86	28.85–17.80
Hk_mock	1,082,100	142.73	148.84	148.57	24.70–11.91
Br_NNV	775,840	145.55	148.17	146.52	31.30–17.80
Br_mock	832,801	147.88	148.08	146.81	17.11–17.78
Active10	80,768	146.81	149.81	149.63	2.04–11.81
Control10	80,768	145.60	140.69	148.17	2.15–11.77
Active50	11,130	145.55	148.80	146.78	2.01–10.45
Control50	11,130	144.62	140.84	149.81	2.06–10.03
Active80	6,862	145.06	150.07	147.88	2.48–12.36
Control80	6,862	142.53	142.34	149.83	2.06–15.12

All models were trained and tested using a cluster, with XGBoost and COMBI SVM using the platform’s runners, those being Intel Xeon Gold architecture CPUs, and DeepCOMBI using NVIDIA A40 48 GB GPUs. CGR used the runner CPUs to generate the images, and then the A40 GPUs to train the neural networks to classify them.

The values listed for CGR are inclusive of both the pre-processing step for creating images and the training of the classifier network, while XGBoost and COMBI use no such pre-processing and work on the raw SNP data. XGBoost always requires around 150 GB of memory due to the way the experiments were set up, first loading the whole genome features and then filtering them to obtain the various datasets, but it is almost always the fastest in terms of time. Combined with its reliability in performance, this makes it a very easy method to apply on any classification task.

COMBI SVM employs the same data loading scheme as XGBoost, resulting in a large amount of memory used, but it is still generally fast, with the caveat of having an upper bound in data processing. The time reported in the table for the Hk datasets is not the time necessary to analyze the whole genome, as that was impossible due to the limitations of the LIBLINEAR library. Instead, we measured the time taken to analyze each chromosome individually by splitting the dataset, to give an estimate of how long it takes to go through that same amount of data. The same data loading is also used on DeepCOMBI, but the training time required for the neural network showcases much larger investment when compared to COMBI SVM, with the results being comparatively more solid in return, but less consistent, with several tests yielding lower performance.

CGR has the lowest memory footprint, pre-processing the data in a lightweight manner and then training a neural network using batches to avoid large memory usage, and results faster overall than DeepCOMBI even with the long pre-processing phase. It performs similarly to DeepCOMBI with results reaching high peaks of performance but being less consistent than XGBoost or COMBI SVM overall.

## 4 Conclusion

In conclusion, this study considered various machine learning methods for SNP and phenotype association, and evaluated their performance on a challenging task of aquaculture genomic data to provide insight on the usefulness of these tools. Predicting a binary trait, such as disease susceptibility, remains an important task for breeding programs. While the test outcomes showcased the difficult nature of this association task, all the tools have shown promising results in at least one scenario, meaning that their performance is very sensitive to the data they are fed.

Ascertaining the performance of the models, under standard testing praxis of an 80% training and 20% test split, the COMBI SVM model obtained the best results, with the downside of being unable to classify the larger Tissue-specific data due to limitation of the software libraries used by the tool. XGBoost closely follows COMBI SVM in its reliability, but both methods struggle to distinguish between the Active and Control datasets, while the DeepCOMBI and CGR methods demonstrated an higher capacity in distinguishing between the two.

Moving on to the two clusters of genomically distant individuals, although the animals did not belong to two independent populations, the scenario proved sufficiently challenging, and the tests were overall far more difficult, with XGBoost remaining the most consistent in terms of classification F1 score. Even with these challenging benchmarks, CGR managed to be a very effective method achieving performance even higher than on the standard 80%–20% tests, although with a power of discrimination between Active and Control that depends on percentage of tissues in which the SNPs occur in the datasets. Interestingly, both the neural network based approaches (DeepCOMBI and CGR) achieved an F1-score in the active dataset that is some decimals bigger than the one obtained with the control dataset. In Active10/Control10 the values are still high, but the difference between the two datasets is lower, while in Active80/Control80 the higher value is reached by the control dataset. Since this is the smallest dataset we believe that this inversion might depend on the fact that neural networks struggles when the training dataset is not big enough. However, this results also give hints on the percentage of tissues in which the active regions need to be present to be able to include a number of SNPs that allows the network training.

Another observation common to both the approaches that use deep learning is that looking at the entire set of experiments their results present high variability. For what concerns DeepCOMBI, the observed high variance in its performance likely depends on sensitivity to hyper-parameters. For CGR we believe that, with longer sequences, the higher density of the points makes the image noisier, making it more difficult for the neural network to recognize patterns.

In terms of computational resources, the memory requirement to analyze the data was the lightest for CGR, although the largest datasets could require nearly a day to process, while conversely XGBoost is generally the fastest tool.

The tools analyzed in this study also enable the assessment of the importance of features or SNPs used during the classification process. Preliminary results confirm that chromosome three contains a major QTL associated with resistance to VNN.

## Data Availability

Publicly available datasets were analyzed in this study. This data can be found here: The datasets analyzed for this study refer to the study Mukiibi et al. (2025) and can be retrieved from the associated repository. Genotypes and phenotypes have been submitted to the Europeans Variant Archive (EVA). Whole-genome raw sequencing data have been uploaded to the NCBI Short Read Archive (SRA) under BioProject accession PRJNA1110973 (https://www.ncbi.nlm.nih.gov/bioproject/PRJNA1110973/). ATAC-seq and ChIP-seq sequencing data, and functional annotation from head kidney samples have been uploaded to the EMBL-EBI repository under accessions PRJEB52284 (https://www.ncbi.nlm.nih.gov/bioproject/PRJEB52284/) and PRJEB59557 (https://www.ncbi.nlm.nih.gov/bioproject/PRJEB59557/), respectively. ATAC-seq and ChIP-seq sequencing data from brain samples have been uploaded to the EMBL-EBI repository under accessions PRJEB52775 (https://www.ncbi.nlm.nih.gov/bioproject/PRJEB52775/) and PRJEB59432 (https://www.ncbi.nlm.nih.gov/bioproject/PRJEB59432/), respectively.

## Footnotes

1 A preliminary version was presented at BIOSTEC-BIOINFORMATICS 2025 (Faldani et al., 2025).
