# Supplementary material for: Predicting VNN resistance in European sea bass using machine learning on high dimensional low sample size data
Source: Front Bioinform. 2026 May 20;6:1718386. doi: 10.3389/fbinf.2026.1718386 (PMC13229855; doi:10.3389/fbinf.2026.1718386)
Supplement: Supplementary file 1 [file DataSheet1.pdf]

Supplementary Material

1 DETAILS OF SOFTWARE TUNING

1.1 Key architectural details of the Neural Networks used with CGR

Network	Input Size	Architecture Details	FC / Dropout	Output Layer	Batch / Epochs
AlexNet	227×227, 1 channel replicated to 3	Conv1: 96 filters, 11×11 kernel, stride 4<br>Conv2: 256 filters, 5×5 kernel, stride 1, padding same<br>Conv3: 384 filters, 3×3 kernel, stride 1, padding same<br>Conv4: 384 filters, 3×3 kernel, stride 1, padding same<br>Conv5: 256 filters, 3×3 kernel, stride 1, padding same<br>MaxPool 3×3 stride 2 after conv1, conv2, conv5; BatchNorm after each conv; ReLU activations	FC1: 4096 neurons, dropout 0.5<br>FC2: 4096 neurons, dropout 0.5	1 neuron, sigmoid	15 / 120
ResNet50	224×224, 1 channel replicated to 3	Conv1: 64 filters, 7×7 kernel, stride 2; MaxPool 3×3 stride 2<br>Stage 2: 1 conv block + 2 identity blocks<br>Stage 3: 1 conv block + 3 identity blocks<br>Stage 4: 1 conv block + 5 identity blocks<br>Stage 5: 1 conv block + 2 identity blocks<br>AvgPool 2×2	– (no additional fully connected layers)	1 neuron, sigmoid	30 / 90
ResNet101	224×224, 1 channel replicated to 3	Conv1: 64 filters, 7×7 kernel, stride 2; MaxPool 3×3 stride 2<br>Stage 2: 1 conv block + 3 identity blocks<br>Stage 3: 1 conv block + 4 identity blocks<br>Stage 4: 1 conv block + 23 identity blocks<br>Stage 5: 1 conv block + 3 identity blocks<br>AvgPool 7×7	– (no additional fully connected layers)	1 neuron, sigmoid	15 / 30

Table S1. Summary of CNN architectures, input preprocessing, and final training parameters for CGR-based classification. All CGR images were resized with bicubic interpolation and replicated on 3 channels; the final dense layer was replaced with a single neuron with sigmoid activation for binary classification. SGD optimizer with learning rate 0.001 was used.

Dataset	Architecture	Epochs	Batch Size	Final Loss	Validation Accuracy
Active 10%	CNN	120	30	0.8176	0.62
Active 10%	ResNet101	30	15	0.6762	0.68
Active 80%	ResNet101	30	15	0.7373	0.65

Table S2. Representative training and validation metrics for the CGR-based model.

1.2 Hyperparameters details for Combi

SVM	L2 regularization with C = 100 and squared hinge loss
COMBI-ML	one hidden layer of 128 neurons; 0.3 dropout rate between each layer; ReLU activation function; L1 and L2 regularization weighted at 0.1 and 0.01 respectively; learning rate 10^{-12} with binary cross-entropy loss and 1000 epochs of training

1.3 Software libraries

The implementations of the methods used in this study relied on the following Python libraries:

- XGBoost approach: experiments were conducted using Python 3.11, with pandas, scikit-learn, numpy, and XGBoost for gradient boosting tree models. Hyperparameters were optimized automatically using Optuna, including number of trees, learning rate, maximum tree depth, minimum child weight, and L1/L2 regularization strengths. The library LazyPredict was used for preliminary benchmarking of multiple models.
- COMBI-SVM: scikit-learn.
- COMBI-ML: TensorFlow with Keras backend.
- CGR approach: experiments were conducted using Python 3.9, with the libraries pandas, matplotlib, scikit-learn, keras, tensorflow, numpy, and Pillow. Additional modules collections and concurrent.futures.ProcessPoolExecutor are part of the Python standard library.

2 XGBOOST GAIN AND COMBI SVM AUC

Chromosome	XGBoost Gain	COMBI SVM AUC
1	206.76	0.5892
2	308.21	0.5411
3	**410.13**	**0.6607**
4	157.75	0.5938
5	97.81	0.5797
6	159.77	0.6194
7	97.41	0.5919
8	94.13	0.5524
9	120.11	0.6292
10	161.38	0.5725
11	240.79	0.5984
12	83.24	0.5766
13	129.82	0.5989
14	114.96	0.5673
15	114.19	0.5343
16	123.57	0.5872
17	64.95	0.533
18	178.83	0.6056
19	147.12	0.5841
20	101.86	0.5709
21	142.77	0.6076
22	86.54	0.5806
23	171.94	0.5775
24	58.92	0.5781

Table S3. XGBoost gain analysis and COMBI SVM chromosome-level AUC analysis. The highest gain/AUC values are highlighted in bold.

Chromosome	Position	Gain
3	10077301	103.13
3	10810942	83.29
3	6521275	44.80
17	3832578	33.58
15	27464293	33.35
11	34067830	30.21
23	7273646	29.24
1	2871406	28.99
11	28584603	27.13
22	14853188	27.06

Table S4. XGBoost gain analysis. The table reports the top 10 SNPs ranked according to their gain.

3 CORRELATIONS BETWEEN TOOLS AND BETWEEN ACTIVE AND CONTROL PREDICTIONS FOR EACH TOOL

datasets	MCC XGBoost's predictions vs DeepCOMBI's predictions on the random split
Hk_NNV_narr	-0.10
Hk_mock_narr	0.07
Br_NNV_narr	0.02
Br_mock_narr	0.07
Active80	-0.05
Control80	-0.09
Active50	-0.02
Control50	-0.08
Active10	-0.08
Control10	0.00

Table S5. Correlation between XGBoost's predictions and DeepCOMBI's predictions with the random split. No correletion between the predictions of the two tools emerged in any of the tested datasets.

MCC random split	XGBoost	COMBI-SVM	DeepCOMBI	CGR
Active80 vs Control80	0.38	0.38	0.03	0.10
Active50 vs Control50	0.44	0.40	0.07	-0,04
Active10 vs Control10	0.21	0.35	-0.01	0.05

Table S6. Correlation between the predictions done by each tool using the Active datasets vs the corresponding prediction obtained using the Control datasets, with the random split. Both XGBoost and SVM show correlation between the predictions obtained using a different feature selection (Active vs Control), meaning their predictions are less affected by the choices of the SNPs.

MCC<br>distant population split	XGBoost	COMBI-SVM	DeepCOMBI	CGR
Active80 vs Control80	0.08	0.51	0.05	0.16
Active50 vs Control50	0.16	0.54	0.03	0.06
Active10 vs Control10	0.08	0.69	-0.05	-0.02

Table S7. Correlation between the predictions done by each tool using the Active datasets vs the correponding prediction obtained using the Control datasets within the distant paopulation split. Only SVM show strong correlation, meaning its predictions are unaffected by the choices of the SNPs.

4 SUMMARY FIGURES

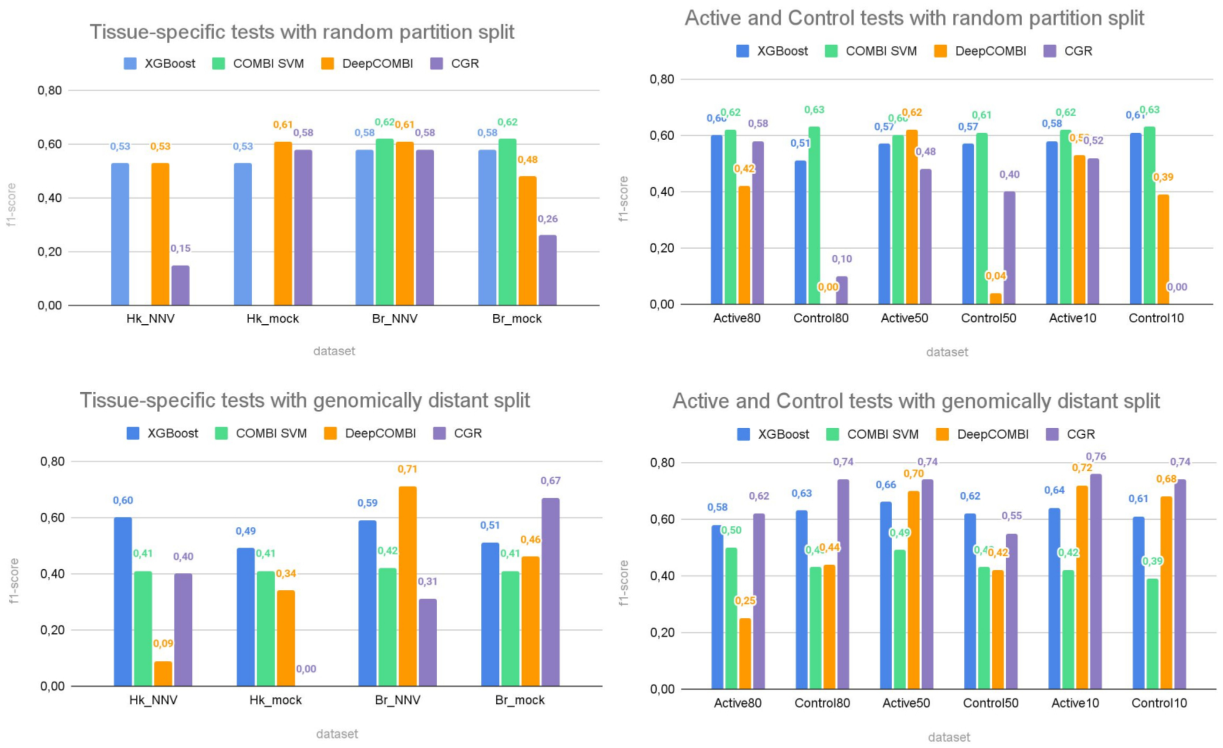

Figure S1. F1-scores for all datasets and splits.
Top-left: F1-scores obtained on Tissue-specific tests using the random partition split. The COMBI SVM approach achieved the highest performance (up to 62%), while DeepCOMBI and CGR reached up to 61% and 58% on Hk_mock and Br_NNV datasets, respectively, and showed reduced performance on Hk_NNV and Br_mock.
Top-right: F1-scores obtained on Active and Control tests using the random partition split. XGBoost discriminates between Active80 and Control80 but shows degraded performance on the 50% and 10% sets, whereas COMBI SVM is unable to distinguish between Active and Control datasets. Neural network-based methods exhibit larger F1-score gaps between Active and Control.
Bottom-left: F1-scores obtained on the Tissue-specific tests using a genomically distant split. High F1-scores are observed in specific cases (60% on Hk_NNV by XGBoost, 71% on Br_NNV by DeepCOMBI, and 67% on Br_mock by CGR). The Hk_mock dataset is challenging for all methods, and COMBI SVM performs poorly across Tissue-specific datasets.
Bottom-right: F1-scores obtained on Active and Control tests using the genomically distant split. All methods except COMBI SVM struggle on the 80% sets, while 50% sets show clear gaps between Active and Control. On the 10% sets, differences are small but consistently favour Active. Overall, the CGR model outperforms the others on Active sets, often reaching or exceeding 70% F1-score.
